# Training of staff within randomised controlled trials (RCTS) of health-care interventions: a systematic review of the literature

**DOI:** 10.1186/1745-6215-16-S2-P186

**Published:** 2015-11-16

**Authors:** Athanasia Gravani, Alexandra Nicholson, Athene Lane, Chris Rogers

**Affiliations:** 1 University of Bristol, Bristol, UK

## Introduction

It is imperative for staff involved in clinical trials to receive appropriate education and training relating to their particular roles to be able to fulfil GCP requirements and ensure successful trial conduct (ASCO, J Oncol Pract. 2008). However, little is known about the ways training is being provided to trial staff.

## Aims

To investigate and summarize training methods currently used in RCTs of health-care interventions.

## Methods

A systematic review was undertaken to identify articles reporting methods and practices of staff training within RCTs. A set of codes was used for the systematic organisation of extracted data.

## Results

7471 records were screened and 89 studies were identified; 78 (88%) focused on training in an individual RCT and 11 (12%) were network-based. Studies were mainly conducted in USA (55%) and internationally (27%). Most were multi-centre (94%) and funded by non-commercial funders (70%). Different combinations of live (face-to-face and remote) and recorded (text-based and multimedia) training methods were used. Training was mainly provided at a group-level (51%), by trainers from the research team (65%). Intervention delivery/fidelity (49%), protocol and trial-related procedures (39%), trial data (36%), patient recruitment (31%) and outcome assessment (18%) were the top five training objectives. Evaluation information was present in 88% of studies, however, a detailed evaluation was only given in 12% of them. Information on the cost of training was only reported in 4.5% of studies.

## Conclusions

There is substantial variability in the reported training process across RCTs of various intervention types, disease areas and sample sizes.

